# Mathematical prediction of the time evolution of the COVID-19 pandemic in Italy by a Gauss error function and Monte Carlo simulations

**DOI:** 10.1140/epjp/s13360-020-00383-y

**Published:** 2020-04-15

**Authors:** Ignazio Ciufolini, Antonio Paolozzi

**Affiliations:** 1 Dipartimento di Ingegneria dell’Innovazione, University of Salento, Lecce, and Centro Fermi, Rome, Italy; 2 Scuola di Ingegneria Aerospaziale, Sapienza, University of Rome, Rome, Italy

## Abstract

In this paper are presented mathematical predictions on the evolution in time of the number of positive cases in Italy of the COVID-19 pandemic based on official data and on the use of a function of the type of a Gauss error function, with four parameters, as a cumulative distribution function. We have analyzed the available data for China and Italy. The evolution in time of the number of cumulative diagnosed positive cases of COVID-19 in China very well approximates a distribution of the type of the error function, that is, the integral of a normal, Gaussian distribution. We have then used such a function to study the potential evolution in time of the number of positive cases in Italy by performing a number of fits of the official data so far available. We then found a statistical prediction for the day in which the peak of the number of daily positive cases in Italy occurs, corresponding to the flex of the fit, that is, to the change in sign of its second derivative (i.e., the change from acceleration to deceleration), as well as of the day in which a substantial attenuation of such number of daily cases is reached. We have also analyzed the predictions of the cumulative number of fatalities in both China and Italy, obtaining consistent results. We have then performed 150 Monte Carlo simulations to have a more robust prediction of the day of the above-mentioned peak and of the day of the substantial decrease in the number of daily positive cases and fatalities. Although official data have been used, those predictions are obtained with a heuristic approach since they are based on a statistical approach and do not take into account either a number of relevant issues (such as number of daily nasopharyngeal swabs, medical, social distancing, virological and epidemiological) or models of contamination diffusion.

## Introduction

**Fig. 1:**
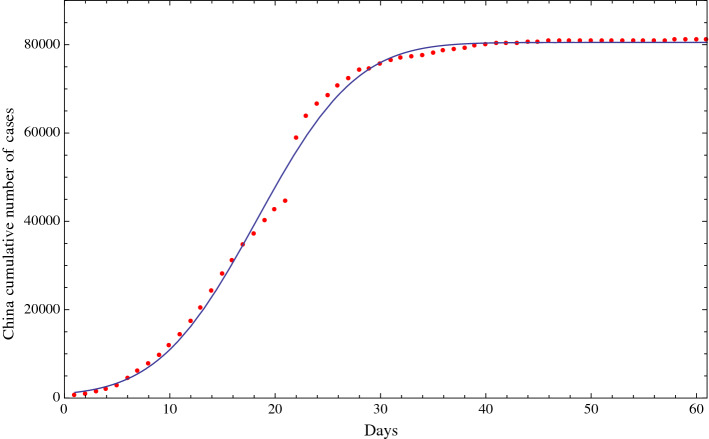
Fit of the cumulative number of diagnosed positive cases of COVID-19 in China (red dots) from January 22, 2020 (included), to March 27, 2020 (included), and the fitting function of the type of a Gauss error function with four parameters (solid line). The horizontal axis reports the days from January 22, 2020; the vertical axis reports the cumulative number of diagnosed positive cases

By considering the cumulative diagnosed positive cases of COVID-19 infections and fatalities available in the Web site of the Italian “Ministero della Salute” [1], Worldometer [2] and World Health Organization [3], we found that they can be well approximated by a cumulative distribution function (CDF) with four parameters of the type of the Gauss error function, that is, the integral of a normal, Gaussian distribution (see Sect. 2). By positive cases, we mean the positive cases actually diagnosed plus, for future days, the positive cases that we expect to be diagnosed. Indeed, it is well known among the virologists that the actual number of positive cases is much higher than the diagnosed ones [4]. However, it is assumed that the diagnosed cases are a good statistical representation of the entire population of the positive cases. In Fig. 1, we report the result of our fit of the cumulative diagnosed positive cases in China. We have then applied such a CDF to study the evolution in time of the number of positive cases in Italy, in the attempt to possibly, statistically, predict the peak in the number of daily positive cases and the possible date of a substantial decrease in the number of daily positive cases. Furthermore, we have also applied our method to the CDF of the number of fatalities in both China and Italy, confirming our predictions obtained with the cumulative positive cases. Finally, in Sect. 4, we have performed a number of Monte Carlo simulations to get a more robust prediction of both the date of the peak in the number of positive cases, diagnosed each day, and in the date after which the number of new positive cases will be below a certain threshold [5].

## Fit of cumulative diagnosed positive cases and fatalities of COVID-19 in China

**Fig. 2:**
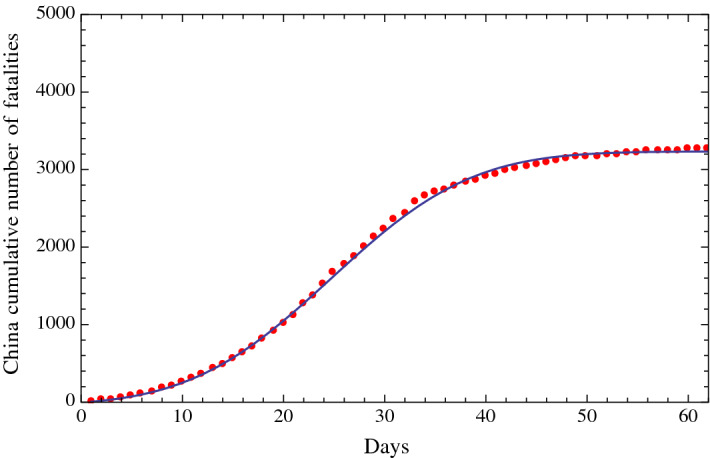
Fit of the cumulative number of fatalities due to COVID-19 in China (red dots) from January 22, 2020 (included), to March 28, 2020 (included), and the fitting function of the type of a Gauss error function with four parameters (solid line). The horizontal axis reports the days from January 22, 2020; the vertical axis reports the cumulative number of fatalities in China

Based on the number of diagnosed positive cases and fatalities of COVID-19 in China, we have fitted the cumulative numbers with a function of the type of the Gauss error function

1 $$\begin{aligned} a+b\,\, \text{ erf }\left( {cx-d}\right) \end{aligned}$$

containing the four parameters *a*, *b*, *c*, *d*,  that we have fitted using the available official data. A similar distribution is also observed in other studies of seasonal influenza and pandemic [6, 7]. The result of the fit is reported in Fig. 1, which shows the good level of the fit using function (1) with those four parameters. In Fig. 2, we report the fit of the cumulative number of fatalities due to COVID-19 in China, which also shows the good level of the fit using function (1). In Sect. 3, the same procedure was applied to the Italian data.

## Fit and predictions of cumulative positive cases and fatalities of COVID-19 in Italy

**Fig. 3:**
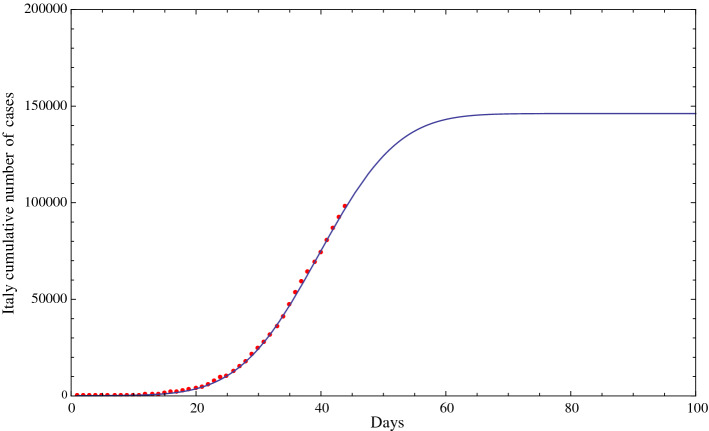
Fit of the cumulative number of diagnosed positive cases of COVID-19 in Italy (red dots) from February 15, 2020 (included), to March 29, 2020 (included), using a fitting function of the type of the Gauss error function with four parameters (solid line). A similar result was obtained with a fitting function of the type of the logistic function

In this section, we report the results of the fit of the cumulative diagnosed positive cases of COVID-19 in Italy using a function of the type of the Gauss error function, given in Sect. 2. We obtained very similar results using a logistic function with four parameters, which are not reported here for the sake of brevity. Figure 3 shows the fit of the data from February 15, 2020, to March 29, 2020. According to this fit, the flex, i.e., the point where the second derivative of the fit is becoming negative, that is, the difference between two successive daily cases becomes negative, or in other words the point where there is a deceleration in the number of positive cases is reached at March 25, 2020, plus or minus 2 days (2-sigma), this uncertainty is derived in Sect. 4. According to this fit, the date of a substantial reduction in the number of cumulative positive cases in Italy (about 100 cases), is April 22, 2020, plus or minus 4.6 days (2-sigma) (see Sect. 4).

**Fig. 4:**
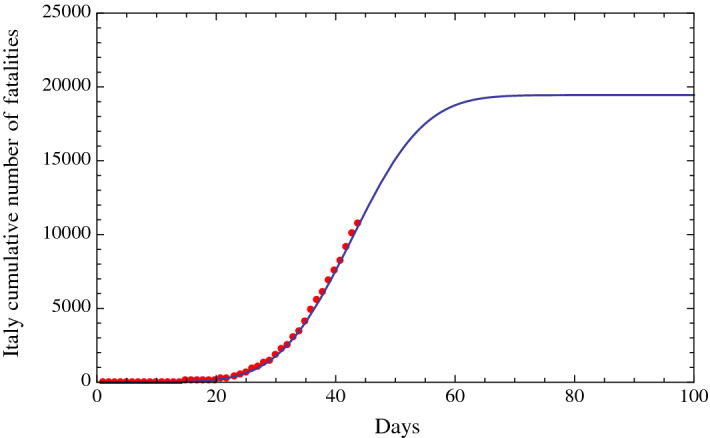
Fit of the cumulative number of fatalities due to COVID-19 in Italy (red dots) from February 15, 2020 (included), to March 29, 2020 (included), using a fitting function of the type of the Gauss error function with four parameters (solid line). The analysis of the curve shows that the date of the flex and the date of a substantial reduction in the number of fatalities are four days after the predictions based on the cumulative number of diagnosed positive cases of COVID-19 in Italy (Fig. 3), thus confirming the consistency of our results

To check whether we obtain the same predictions using the cumulative number of fatalities of COVID-19 in Italy, instead of using the number of positive cases of COVID-19 in Italy, we repeated the previous analysis using the cumulative fatalities enumerated in the Web sites of the Italian “Ministero della Salute” [1], Worldometer [2] and World Health Organization [3]. The results are shown in Fig. 4. The analysis of the curve shows that the flex and the number of a substantial reduction in the number of fatalities (about 11 fatalities, i.e., approximately proportional to the threshold of 100 positive cases, chosen in the previous analysis, times the ratio of fatalities to positive cases) are both only four days after the previous predictions, thus confirming the consistency of our results using the positive cases.

To evaluate the standard deviation relative to the date of the flex, we have used two methods. The first one is to fit the cumulative diagnosed positive cases in Italy using the data from the beginning, February 15, 2020 (included), to March 16, 2020 (included), then from February 15, 2020, to March 17, 2020, and so on, until March 29, 2020 (included), thus getting 14 evaluations of the date of the flex. We then evaluated the standard deviation of these 14 points, and we obtained a 1-sigma standard deviation for *the flex* of 3 days. The 2-sigma (95.5% probability) and 3-sigma (99.7% probability) uncertainties in the day of the flex are then 6 days and 9 days, respectively. In regard to *the date of a substantial reduction in the number of cumulative positive cases* in Italy (about 100 cases), the standard deviation is 6 days (68.2% probability), 12 days (95.5% probability) and 18 days (99.7% probability). Both the date of the flex and the date of a substantial reduction in the number of cases have a quasi oscillating behavior whose amplitude is decreasing by increasing the final date of the analysis, i.e., increasing the number of points used in the fit. Indeed, using the last seven evaluations out of the 14 ones, the previous standard deviations reduce by a factor of about two. However, using a second more robust method, by a Monte Carlo analysis [8–10], we evaluated narrower uncertainties for the day of the flex and for the day of a substantial reduction in the number of diagnosed positive cases, as described in Sect. 4.

## Monte Carlo simulations of cumulative positive cases of COVID-19 in Italy

The Monte Carlo simulations [8–10] have been designed to possibly take into account the measurement error in each daily number of the cumulative positive cases of COVID-19 in Italy. This error should describe the uncertainty in the process of measuring the daily number of positive cases due to fluctuations in the measurement procedures (such as a different number of performed daily nasopharyngeal swabs of one day with respect to another day); of course, this error does not describe the difference between the actual total positive cases and the diagnosed ones which can be very large [4]. However, the diagnosed cases are hypothesized to be a representative sample of the actual population (i.e., of the total number of positive cases, which is unknown). To get an estimate of the uncertainty in each daily number, we applied the following heuristic approach. We have assumed a measurement uncertainty in the total number of positive cases equal to 10% of each daily number (Gaussian distributed).

The second step was to generate a random matrix $\left( {m\times n} \right)$, where *n* (columns) is the number of observed days and *m* (rows) is the number of random outcomes, which we have chosen to be 150. Each number in the matrix is part of a Gaussian distribution with mean equal to 1 and sigma equal to 0.1 (i.e., 10% of 1), both row-wise and column-wise. In such a way, each day will be characterized by 150 simulated outcomes that allow to apply a statistical approach. The 150 outcomes represent a reasonably large number of simulated deviations from the official data.

We then multiplied the nominal value of the diagnosed positive cases of the *j*th day for the 150 numbers of the *j*th column of the random matrix mentioned above. In such a way, each day will be associated with a series of 150 numbers (Gaussian distributed with a 10% standard deviation), which simulate the statistical nature of a single datum. The index *j* will run from February 15, 2020, to March 26, 2020. Finally, we integrated the daily data to obtain 150 series of the cumulative diagnosed positive cases that allowed to perform the statistical analysis.

**Fig. 5:**
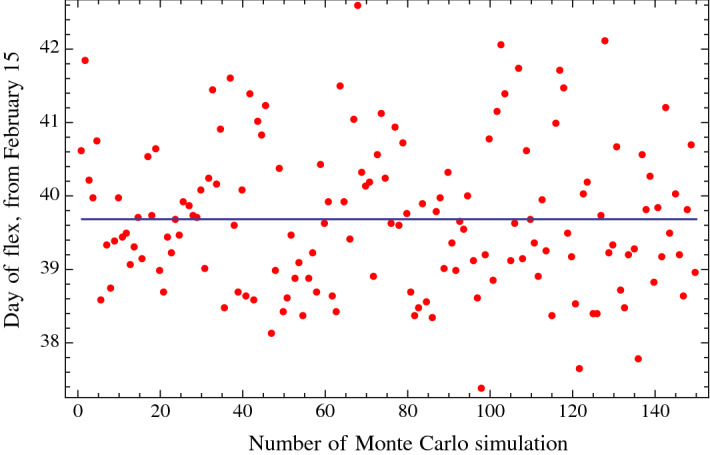
Monte Carlo simulations: each red dot corresponds to the day of occurrence of the flex (after which there is a reduction in the number of daily cases, i.e., there is a deceleration in the number of daily cases), obtained with each of the 150 Monte Carlo simulations. The vertical axis reports the number of days from February 15, 2020

**Fig. 6:**
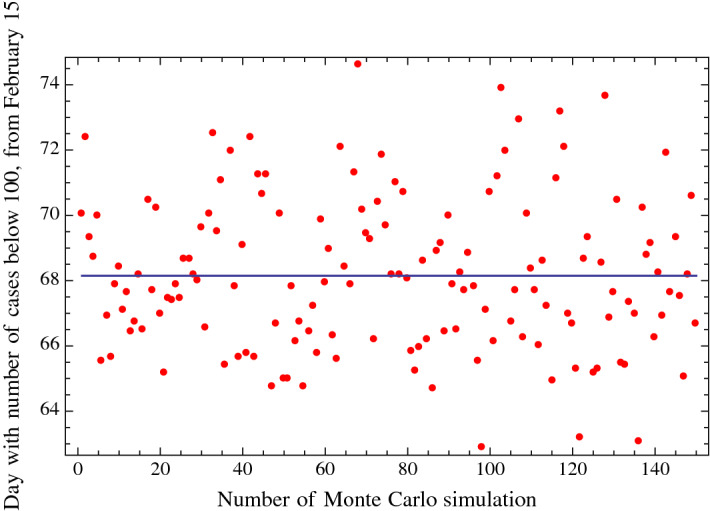
Monte Carlo simulations: each red dot corresponds to the day in which a substantial reduction in the number of daily cases (about 100) occurs. Each red dot is obtained with each of the 150 Monte Carlo simulations. The vertical axis reports the number of days from February 15, 2020

In summary, starting from the *n* nominal values of the daily data, we generated *n* Gaussian distributions with 150 outcomes, with mean equal to the *n* nominal values and with 10% standard deviation. Then, for each of the 150 simulations, these *n* values (corresponding to the cumulative positive cases of *n* days) were fitted with a four-parameter function of the type of the Gauss error function, and we then determined the date of the flex with such fitted function for each simulation. Using the fitted function, we also determined the date at which the number of daily positive cases will be at a certain threshold that, for example, we have chosen to be 100. Finally, we calculated the standard deviation of these 150 simulations. In Fig. 5 and Fig. 6, we report the values (red dots) and the mean (horizontal solid line) of the Monte Carlo simulations, respectively, for the date of the flex and for the date of a substantial reduction in the number of daily positive cases.

Using $n = 41\hbox { days}$ (i.e., the number of daily diagnosed positive cases up to March 26, 2020), the mean of the 150 Monte Carlo simulations gives the expected dates of March 25, 2020, and April 22, 2020, for the flex and the day of a substantial reduction in the number of daily cases (about 100), respectively. We then obtained a standard deviation (1-sigma) of 1 day for the date of the flex and of 2.3 days for the date in which a substantial reduction in the number of daily cases would be about 100. This result corresponds to a probability of 68.2% that the date of the flex will be at a certain date plus or minus 1 day and that the date of a substantial reduction in the number of cases will be at a certain date plus or minus 2.3 days. A 2-sigma standard deviation will give a more robust probability of 95.4% of the day of the flex and of the day of a substantial reduction in the number of cases. The 2-sigma values correspond to plus or minus 2 days for the day of the flex and plus or minus 4.6 days for the day of a substantial reduction in the number of cases. A similar uncertainty was obtained for the day of the flex and the day of a substantial reduction in the number of fatalities.

## Conclusions

In this paper, we considered the first 44 days for fitting the cumulative diagnosed positive cases of COVID-19 in Italy (i.e., from February 15, 2020, to March 29, 2020) and the first 41 days for the Monte Carlo simulations. The function used for the fitting is of the type of the Gauss error function with four parameters (a distribution function which well fits the corresponding cumulative diagnosed positive cases in China). We obtained that the day of the flex (i.e., the day of deceleration in the number of daily positive cases) is in Italy, with a 95.4% probability, between March 23, 2020, and March 27, 2020; the 2-sigma uncertainty of $+/-$ 2 days was obtained with Monte Carlo simulations. In regard to the day of a substantial reduction in the number of daily positive cases (which, for example, we took to be 100), this day will be in Italy, with a 95.4% probability, between April 17, 2020, and April 27, 2020; the 2-sigma uncertainty of $+/-$ 4.6 days was also obtained with the Monte Carlo simulations. In regard to the day of the flex and the day of a substantial reduction in the number of fatalities, we obtained a four-day forward shift with respect to the corresponding dates of the positive cases. The predictions discussed in this paper are statistical in nature and do not explicitly take into account the relevant factors of the daily number of nasopharyngeal swabs, social distancing, and epidemiological and virology studies, which are outside the analysis of the present paper.

It is important to stress that the mathematical predictions reported in this paper provide an indication of the approximate period when a substantial reduction of positive cases and fatalities is expected and not the end of the extremely important mitigation measures.
